# Bis(μ-4-bromo­benzoato)-κ^3^ *O*,*O*′:*O*′;*O*:*O*,*O*′-bis­[μ-1,3-bis­(pyridin-4-yl)propane-κ^2^ *N*:*N*′]bis­[(4-bromo­benzoato-κ^2^ *O*,*O*′)cadmium]

**DOI:** 10.1107/S1600536811043996

**Published:** 2011-10-29

**Authors:** Dong Liu, Yan-Qin Zi, Seik Weng Ng

**Affiliations:** a College of Chemistry and Materials Science, Huaibei Normal University, Huaibei 235000, Anhui, People’s Republic of China; b Department of Chemistry, University of Malaya, 50603 Kuala Lumpur, Malaysia; c Chemistry Department, Faculty of Science, King Abdulaziz University, PO Box 80203 Jeddah, Saudi Arabia

## Abstract

The dinuclear complex, [Cd_2_(C_7_H_4_BrO_2_)_4_(C_13_H_14_N_2_)_2_], lies on a twofold rotation axis crossing midway between the two metal atoms. The Cd^II^ cation is seven-coordinated with a geometry that can be considered as distorted penta­gonal bipyramidal, with the N atom of the *N*-heterocyclic units occupying the apical sites and the O atoms of the 4-bromo­benzoate units in the equatorial plane. The middle methyl­ene group of the 1,3-bis­(4-pyrid­yl)propane ligands is located outside of the twofold rotation axis and consequently is disordered over two sites around this symmetry element with fixed occupancies factors of 0.5.

## Related literature

For related structures, see: Liu *et al.* (2011 ▶). For another complex with a dinuclear seven-coordinate Cd(II) atom, see: Ranjbar *et al.* (2002 ▶); Wang *et al.* (2006 ▶). 
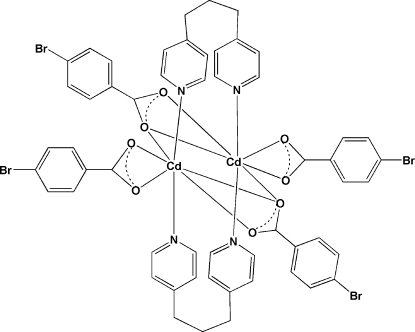


## Experimental

### 

#### Crystal data

[Cd_2_(C_7_H_4_BrO_2_)_4_(C_13_H_14_N_2_)_2_]
*M* *_r_* = 1421.35
Tetragonal, 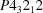

*a* = 13.7829 (10) Å
*c* = 27.8235 (17) Å
*V* = 5285.6 (5) Å^3^
*Z* = 4
Mo *K*α radiation
μ = 3.89 mm^−1^
*T* = 223 K
0.45 × 0.40 × 0.35 mm

#### Data collection

Rigaku Mercury area-detector diffractometer
Absorption correction: multi-scan (*REQAB*; Jacobson, 1998 ▶) *T*_min_ = 0.174, *T*_max_ = 0.256
25017 measured reflections
6020 independent reflections
5452 reflections with *I* > 2σ(*I*)
*R*_int_ = 0.051

#### Refinement

*R*[*F* ^2^ > 2σ(*F* ^2^)] = 0.044
*wR*(*F* ^2^) = 0.104
*S* = 1.05
6020 reflections
334 parameters
34 restraints
H-atom parameters constrained
Δρ_max_ = 0.73 e Å^−3^
Δρ_min_ = −0.71 e Å^−3^
Absolute structure: Flack (1983) ▶, 2533 Friedel pairs
Flack parameter: 0.006 (12)

### 

Data collection: *CrystalClear* (Rigaku, 2001 ▶); cell refinement: *CrystalClear*; data reduction: *CrystalStructure* (Rigaku/MSC, 2004 ▶); program(s) used to solve structure: *SHELXS97* (Sheldrick, 2008 ▶); program(s) used to refine structure: *SHELXL97* (Sheldrick, 2008 ▶); molecular graphics: *X-SEED* (Barbour, 2001 ▶); software used to prepare material for publication: *publCIF* (Westrip, 2010 ▶).

## Supplementary Material

Crystal structure: contains datablock(s) I, global. DOI: 10.1107/S1600536811043996/lr2033sup1.cif

Structure factors: contains datablock(s) I. DOI: 10.1107/S1600536811043996/lr2033Isup2.hkl

Additional supplementary materials:  crystallographic information; 3D view; checkCIF report

## Figures and Tables

**Fig. 1.**
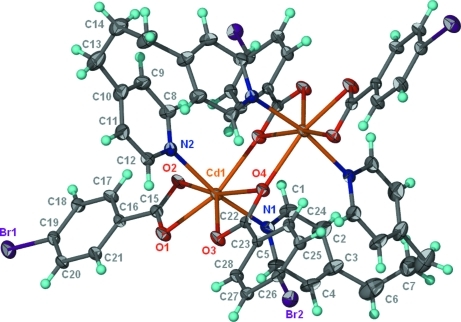
Coordination environment of CdII with non-hydrogen atoms represented by thermal ellipsoids drawn at the 30% probability level;, hydrogen atoms are drawn as spheres of arbitrary radius. The unlabeled atoms are related to the labeled ones by y, x, 1 - z.

## supplementary crystallographic information

### Comment

The crystal structures of coordination compounds having flexible *N*-donor ligands are generally different from those having rigid *N*-donor ligands because these flexible ligands can adopt more than one conformation. The title adduct, [Cd_2_(C_7_H_4_O_2_Br)_4_(C_13_H_14_N_2_)_2_] (Scheme I), is assembled from Cd(II) bis(4-bromobenzoate) and 1,3-bis(4-pyridyl)propane. This study represents an extension of our previous one (Liu *et al.*, 2011).

The complex, which features two Cd^II^ atoms, is lying on a twofold crystallographic rotation axis crossing midway between the two metallic atoms. Each Cd^II^ atom is seven coordinated with an geometry that can be considered as a distorted pentagonal bipyramid, with the nitrogen atom of the N-heterocycle units occupying the apical sites and the oxygen atoms of the p-bromobenzoate units in the ecuatorial plane of each coordination bypiramide (Fig. 1). The middle methylene groups of the 1,3-bis(4-pyridyl)propane ligands are located outside of the binary crystallographic axis and consequently are disordered between two sites around this symmetry element with fixed occupancies factors of 0.5.

### Experimental

To a 10 mL Pyrex glass tube was loaded Cd(NO_3_)_2_.4H_2_O (31 mg, 0.1 mmol), 4-bromobenzoic acid (40 mg, 0.2 mmol), 1,3-bis(4-pyridyl)propane (20 mg, 0.1 mmol) and 3 ml of water. The tube was sealed and heated in an oven to 423 K for three days. It was then cooled to ambient temperature at the rate of 5 K h^-1^ to yield colorless crystals. These were washed with water/ethanol and dried; yield: 54 mg (76% yield based on Cd). Analysis: Calcd. for C_54_H_44_Br_4_Cd_2_N_4_O_8_: C, 45.63; H, 3.12; N, 3.94%. Found: C, 45.83; H, 2.94; N, 3.88%.

### Refinement

Hydrogen atoms were placed in geometrically idealized positions (C–H = 0.95–0.98 Å) and were constrained to ride on their parent atoms with *U*_iso_(H) = 1.2–1.5*U*_eq_(C). The two half-molecules of the *N*-heterocycle have half-occupancy for their middle methylene carbon. For the —CH_2_—CH_2_—CH_2_— linkage, the 1,2-related distances were restrained to 1.54±0.01 Å and the 1,3-related ones to 2.51±0.01 Å. The anisotropic temperature factors of the methylene carbon atoms were restrained to be nearly istropic.

### Crystal data

**Table:**

[Cd_2_(C_7_H_4_BrO_2_)_4_(C_13_H_14_N_2_)_2_]	*D*_x_ = 1.786 Mg m^−^^3^
*M**_r_* = 1421.35	Mo *K*α radiation, λ = 0.71073 Å
Tetragonal, *P*4_3_2_1_2	Cell parameters from 18739 reflections
Hall symbol: P 4nw 2abw	θ = 3.0–27.5°
*a* = 13.7829 (10) Å	µ = 3.89 mm^−^^1^
*c* = 27.8235 (17) Å	*T* = 223 K
*V* = 5285.6 (5) Å^3^	Block, colorless
*Z* = 4	0.45 × 0.40 × 0.35 mm
*F*(000) = 2784	

### Data collection

**Table:**

Rigaku Mercury area-detector diffractometer	6020 independent reflections
Radiation source: fine-focus sealed tube	5452 reflections with *I* > 2σ(*I*)
graphite	*R*_int_ = 0.051
ω scans	θ_max_ = 27.5°, θ_min_ = 3.0°
Absorption correction: multi-scan (REQAB; Jacobson, 1998)	*h* = −17→15
*T*_min_ = 0.174, *T*_max_ = 0.256	*k* = −14→17
25017 measured reflections	*l* = −36→34

### Refinement

**Table:**

Refinement on *F*^2^	Secondary atom site location: difference Fourier map
Least-squares matrix: full	Hydrogen site location: inferred from neighbouring sites
*R*[*F*^2^ > 2σ(*F*^2^)] = 0.044	H-atom parameters constrained
*wR*(*F*^2^) = 0.104	*w* = 1/[σ^2^(*F*_o_^2^) + (0.0507*P*)^2^] where *P* = (*F*_o_^2^ + 2*F*_c_^2^)/3
*S* = 1.05	(Δ/σ)_max_ = 0.001
6020 reflections	Δρ_max_ = 0.73 e Å^−^^3^
334 parameters	Δρ_min_ = −0.71 e Å^−^^3^
34 restraints	Absolute structure: Flack (1983), 2533 Friedel pairs
Primary atom site location: structure-invariant direct methods	Flack parameter: 0.006 (12)

### Special details

**Table:**

Geometry. All e.s.d.'s (except the e.s.d. in the dihedral angle between two l.s. planes) are estimated using the full covariance matrix. The cell e.s.d.'s are taken into account individually in the estimation of e.s.d.'s in distances, angles and torsion angles; correlations between e.s.d.'s in cell parameters are only used when they are defined by crystal symmetry. An approximate (isotropic) treatment of cell e.s.d.'s is used for estimating e.s.d.'s involving l.s. planes.

### Fractional atomic coordinates and isotropic or equivalent isotropic displacement parameters (Å^2^)

**Table:**

	*x*	*y*	*z*	*U*_iso_*/*U*_eq_	Occ. (<1)
Cd1	0.28537 (3)	0.30187 (3)	0.571507 (12)	0.03592 (10)	
Br1	0.31290 (6)	0.12444 (5)	0.87743 (2)	0.0635 (2)	
Br2	−0.11352 (5)	0.71348 (5)	0.41927 (2)	0.05446 (17)	
N1	0.4148 (3)	0.4080 (3)	0.56841 (16)	0.0429 (11)	
N2	0.1775 (3)	0.1737 (3)	0.56787 (15)	0.0396 (10)	
O1	0.2543 (3)	0.3183 (3)	0.65343 (12)	0.0503 (10)	
O2	0.3615 (3)	0.2054 (3)	0.63554 (12)	0.0456 (9)	
O3	0.1690 (3)	0.4235 (3)	0.56093 (13)	0.0482 (10)	
O4	0.2200 (3)	0.3720 (3)	0.49044 (12)	0.0427 (8)	
C1	0.5048 (4)	0.3806 (5)	0.5593 (3)	0.0567 (16)	
H1	0.5180	0.3146	0.5568	0.068*	
C2	0.5805 (5)	0.4457 (5)	0.5532 (3)	0.072 (2)	
H2	0.6428	0.4229	0.5470	0.086*	
C3	0.5639 (5)	0.5433 (5)	0.5562 (3)	0.072 (2)	
C4	0.4708 (6)	0.5709 (5)	0.5675 (3)	0.071 (2)	
H4	0.4562	0.6365	0.5707	0.086*	
C5	0.3986 (5)	0.5025 (4)	0.5741 (3)	0.0571 (17)	
H5	0.3368	0.5232	0.5827	0.069*	
C6	0.6408 (7)	0.6190 (7)	0.54516 (18)	0.112 (3)	
H6A	0.6119	0.6825	0.5497	0.135*	
H6B	0.6920	0.6125	0.5689	0.135*	
C7	0.6851 (8)	0.6173 (12)	0.4982 (3)	0.097 (6)	0.50
H7A	0.7127	0.5533	0.4933	0.117*	0.50
H7B	0.7384	0.6633	0.4983	0.117*	0.50
C8	0.2078 (4)	0.0851 (4)	0.5583 (2)	0.0527 (15)	
H8	0.2742	0.0747	0.5554	0.063*	
C9	0.1461 (5)	0.0068 (5)	0.5524 (3)	0.0592 (18)	
H9	0.1707	−0.0550	0.5468	0.071*	
C10	0.0476 (4)	0.0221 (5)	0.5549 (3)	0.0595 (17)	
C11	0.0170 (4)	0.1122 (5)	0.5678 (3)	0.0570 (16)	
H11	−0.0487	0.1237	0.5730	0.068*	
C12	0.0826 (4)	0.1862 (5)	0.5731 (2)	0.0484 (14)	
H12	0.0596	0.2478	0.5807	0.058*	
C13	−0.0237 (5)	−0.0588 (5)	0.54387 (18)	0.085 (3)	
H13A	−0.0101	−0.1120	0.5657	0.102*	
H13B	−0.0882	−0.0352	0.5515	0.102*	
C14	−0.0264 (11)	−0.0976 (7)	0.4959 (3)	0.079 (4)	0.50
H14A	−0.0705	−0.1524	0.4954	0.095*	0.50
H14B	0.0377	−0.1216	0.4878	0.095*	0.50
C15	0.3081 (4)	0.2494 (4)	0.66474 (18)	0.0375 (11)	
C16	0.3087 (3)	0.2182 (4)	0.71718 (16)	0.0332 (10)	
C17	0.3666 (4)	0.1423 (4)	0.7316 (2)	0.0450 (13)	
H17	0.4041	0.1095	0.7090	0.054*	
C18	0.3697 (5)	0.1144 (4)	0.7793 (2)	0.0470 (14)	
H18	0.4103	0.0645	0.7895	0.056*	
C19	0.3100 (4)	0.1637 (4)	0.81186 (18)	0.0410 (12)	
C20	0.2518 (4)	0.2390 (4)	0.79782 (18)	0.0432 (13)	
H20	0.2134	0.2712	0.8202	0.052*	
C21	0.2507 (4)	0.2665 (4)	0.7503 (2)	0.0405 (11)	
H21	0.2111	0.3173	0.7403	0.049*	
C22	0.1681 (4)	0.4273 (4)	0.51581 (17)	0.0327 (10)	
C23	0.1022 (3)	0.4995 (3)	0.49208 (17)	0.0314 (10)	
C24	0.1013 (4)	0.5092 (4)	0.44265 (18)	0.0380 (11)	
H24	0.1431	0.4716	0.4241	0.046*	
C25	0.0382 (4)	0.5750 (4)	0.41988 (19)	0.0409 (12)	
H25	0.0379	0.5822	0.3866	0.049*	
C26	−0.0224 (4)	0.6278 (4)	0.4483 (2)	0.0408 (12)	
C27	−0.0225 (4)	0.6223 (4)	0.4981 (2)	0.0415 (12)	
H27	−0.0638	0.6609	0.5164	0.050*	
C28	0.0419 (4)	0.5564 (4)	0.52030 (19)	0.0408 (12)	
H28	0.0439	0.5512	0.5536	0.049*	

### Atomic displacement parameters (Å^2^)

**Table:**

	*U*^11^	*U*^22^	*U*^33^	*U*^12^	*U*^13^	*U*^23^
Cd1	0.0413 (2)	0.0398 (2)	0.02666 (17)	0.00572 (16)	−0.00150 (14)	0.00022 (14)
Br1	0.0817 (5)	0.0768 (4)	0.0321 (3)	0.0083 (4)	−0.0002 (3)	0.0118 (3)
Br2	0.0561 (3)	0.0527 (3)	0.0546 (3)	0.0111 (3)	−0.0103 (3)	0.0124 (3)
N1	0.045 (2)	0.044 (2)	0.040 (2)	0.002 (2)	−0.001 (2)	−0.009 (2)
N2	0.039 (2)	0.043 (2)	0.037 (2)	0.0020 (19)	−0.0006 (19)	0.003 (2)
O1	0.064 (3)	0.055 (2)	0.0320 (19)	0.023 (2)	0.0008 (17)	0.0043 (17)
O2	0.051 (2)	0.057 (2)	0.0297 (18)	0.014 (2)	0.0039 (16)	−0.0019 (17)
O3	0.057 (2)	0.061 (2)	0.0266 (19)	0.0199 (19)	0.0005 (18)	0.0005 (18)
O4	0.050 (2)	0.045 (2)	0.0339 (18)	0.0106 (18)	0.0011 (17)	0.0015 (16)
C1	0.049 (3)	0.045 (3)	0.076 (5)	0.006 (3)	−0.007 (3)	−0.013 (3)
C2	0.038 (3)	0.062 (4)	0.115 (7)	−0.004 (3)	−0.005 (4)	−0.002 (4)
C3	0.061 (4)	0.050 (4)	0.106 (6)	−0.015 (3)	−0.021 (4)	−0.006 (4)
C4	0.076 (5)	0.035 (3)	0.102 (6)	0.001 (3)	−0.011 (5)	−0.011 (4)
C5	0.061 (4)	0.046 (3)	0.064 (4)	0.008 (3)	−0.005 (3)	−0.020 (3)
C6	0.087 (6)	0.080 (5)	0.170 (8)	−0.017 (5)	−0.041 (6)	0.005 (6)
C7	0.083 (8)	0.091 (9)	0.118 (10)	−0.022 (7)	0.011 (8)	−0.012 (8)
C8	0.040 (3)	0.045 (3)	0.073 (4)	0.003 (3)	−0.006 (3)	−0.006 (3)
C9	0.047 (3)	0.045 (3)	0.085 (5)	0.007 (3)	0.009 (3)	−0.006 (3)
C10	0.044 (3)	0.056 (4)	0.078 (5)	−0.006 (3)	0.004 (3)	0.008 (4)
C11	0.041 (3)	0.056 (4)	0.075 (5)	−0.002 (3)	0.018 (3)	0.000 (4)
C12	0.046 (3)	0.048 (3)	0.050 (3)	0.003 (3)	0.009 (3)	−0.005 (3)
C13	0.054 (4)	0.062 (4)	0.140 (7)	−0.012 (4)	−0.001 (5)	0.017 (5)
C14	0.078 (8)	0.063 (7)	0.097 (9)	−0.023 (6)	0.005 (7)	−0.010 (7)
C15	0.037 (2)	0.041 (3)	0.034 (3)	0.001 (2)	−0.003 (2)	−0.001 (2)
C16	0.033 (2)	0.041 (2)	0.025 (2)	0.005 (2)	−0.0024 (19)	0.000 (2)
C17	0.052 (3)	0.047 (3)	0.036 (3)	0.017 (3)	−0.003 (2)	−0.003 (2)
C18	0.062 (4)	0.043 (3)	0.036 (3)	0.010 (3)	−0.004 (3)	0.007 (2)
C19	0.047 (3)	0.047 (3)	0.029 (2)	−0.002 (3)	−0.002 (2)	0.004 (2)
C20	0.049 (3)	0.048 (3)	0.033 (3)	0.008 (3)	0.007 (2)	0.000 (2)
C21	0.043 (3)	0.042 (3)	0.036 (3)	0.012 (2)	0.002 (2)	0.002 (2)
C22	0.034 (2)	0.032 (2)	0.032 (3)	−0.0013 (19)	0.002 (2)	0.002 (2)
C23	0.035 (3)	0.029 (2)	0.029 (2)	−0.0025 (19)	0.004 (2)	0.0007 (19)
C24	0.037 (3)	0.043 (3)	0.034 (3)	−0.004 (2)	0.003 (2)	0.000 (2)
C25	0.048 (3)	0.044 (3)	0.030 (3)	−0.001 (2)	−0.002 (2)	0.004 (2)
C26	0.046 (3)	0.032 (3)	0.044 (3)	−0.004 (2)	−0.004 (2)	0.002 (2)
C27	0.044 (3)	0.039 (3)	0.041 (3)	0.004 (2)	−0.001 (2)	0.000 (2)
C28	0.051 (3)	0.042 (3)	0.029 (2)	0.004 (2)	−0.003 (2)	0.000 (2)

### Geometric parameters (Å, °)

**Table:**

Cd1—N1	2.309 (5)	C9—C10	1.375 (9)
Cd1—N2	2.311 (5)	C9—H9	0.9300
Cd1—O1	2.330 (3)	C10—C11	1.360 (9)
Cd1—O3	2.338 (4)	C10—C13	1.518 (7)
Cd1—O4^i^	2.381 (3)	C11—C12	1.371 (9)
Cd1—O2	2.458 (4)	C11—H11	0.9300
Cd1—O4	2.614 (3)	C12—H12	0.9300
Br1—C19	1.903 (5)	C13—C14	1.439 (8)
Br2—C26	1.904 (5)	C13—C14^i^	1.568 (9)
N1—C1	1.323 (7)	C13—H13A	0.9700
N1—C5	1.331 (7)	C13—H13B	0.9700
N2—C8	1.318 (7)	C14—C13^i^	1.568 (9)
N2—C12	1.328 (7)	C14—H14A	0.9700
O1—C15	1.245 (6)	C14—H14B	0.9700
O2—C15	1.253 (6)	C15—C16	1.521 (6)
O3—C22	1.256 (6)	C16—C17	1.376 (7)
O4—C22	1.262 (6)	C16—C21	1.390 (7)
O4—Cd1^i^	2.381 (3)	C17—C18	1.383 (8)
C1—C2	1.386 (9)	C17—H17	0.9300
C1—H1	0.9300	C18—C19	1.400 (8)
C2—C3	1.368 (10)	C18—H18	0.9300
C2—H2	0.9300	C19—C20	1.368 (8)
C3—C4	1.374 (10)	C20—C21	1.376 (7)
C3—C6	1.519 (7)	C20—H20	0.9300
C4—C5	1.383 (10)	C21—H21	0.9300
C4—H4	0.9300	C22—C23	1.500 (7)
C5—H5	0.9300	C23—C24	1.382 (7)
C6—C7	1.441 (8)	C23—C28	1.387 (7)
C6—C7^i^	1.547 (9)	C24—C25	1.408 (8)
C6—H6A	0.9700	C24—H24	0.9300
C6—H6B	0.9700	C25—C26	1.360 (8)
C7—C6^i^	1.547 (9)	C25—H25	0.9300
C7—H7A	0.9700	C26—C27	1.389 (8)
C7—H7B	0.9700	C27—C28	1.412 (7)
C8—C9	1.385 (8)	C27—H27	0.9300
C8—H8	0.9300	C28—H28	0.9300
			
N1—Cd1—N2	168.48 (15)	C10—C9—C8	118.8 (6)
N1—Cd1—O1	96.70 (16)	C10—C9—H9	120.6
N2—Cd1—O1	89.95 (16)	C8—C9—H9	120.6
N1—Cd1—O3	94.06 (16)	C11—C10—C9	117.4 (6)
N2—Cd1—O3	95.80 (15)	C11—C10—C13	121.5 (6)
O1—Cd1—O3	85.85 (13)	C9—C10—C13	121.1 (6)
N1—Cd1—O4^i^	83.45 (15)	C10—C11—C12	120.2 (6)
N2—Cd1—O4^i^	85.92 (15)	C10—C11—H11	119.9
O1—Cd1—O4^i^	147.81 (12)	C12—C11—H11	119.9
O3—Cd1—O4^i^	126.32 (12)	N2—C12—C11	122.8 (6)
N1—Cd1—O2	92.32 (15)	N2—C12—H12	118.6
N2—Cd1—O2	83.84 (14)	C11—C12—H12	118.6
O1—Cd1—O2	54.69 (12)	C14—C13—C10	118.5 (7)
O3—Cd1—O2	140.51 (12)	C14—C13—C14^i^	55.6 (10)
O4^i^—Cd1—O2	93.12 (12)	C10—C13—C14^i^	110.7 (7)
N1—Cd1—O4	89.98 (14)	C14—C13—H13A	107.7
N2—Cd1—O4	91.32 (14)	C10—C13—H13A	107.7
O1—Cd1—O4	138.15 (12)	C14^i^—C13—H13A	141.4
O3—Cd1—O4	52.42 (11)	C14—C13—H13B	107.7
O4^i^—Cd1—O4	73.93 (13)	C10—C13—H13B	107.7
O2—Cd1—O4	166.50 (11)	C14^i^—C13—H13B	57.6
N1—Cd1—C15	96.62 (15)	H13A—C13—H13B	107.1
N2—Cd1—C15	84.99 (15)	C13—C14—C13^i^	114.9 (8)
O1—Cd1—C15	27.28 (14)	C13—C14—H14A	108.5
O3—Cd1—C15	113.01 (14)	C13^i^—C14—H14A	108.5
O4^i^—Cd1—C15	120.56 (14)	C13—C14—H14B	108.5
O2—Cd1—C15	27.50 (13)	C13^i^—C14—H14B	108.5
O4—Cd1—C15	164.60 (14)	H14A—C14—H14B	107.5
C1—N1—C5	117.4 (6)	O1—C15—O2	123.7 (5)
C1—N1—Cd1	123.4 (4)	O1—C15—C16	117.5 (5)
C5—N1—Cd1	119.1 (4)	O2—C15—C16	118.8 (4)
C8—N2—C12	117.1 (5)	O1—C15—Cd1	59.1 (3)
C8—N2—Cd1	120.8 (4)	O2—C15—Cd1	65.0 (3)
C12—N2—Cd1	122.0 (4)	C16—C15—Cd1	173.6 (3)
C15—O1—Cd1	93.6 (3)	C17—C16—C21	120.3 (5)
C15—O2—Cd1	87.5 (3)	C17—C16—C15	119.9 (5)
C22—O3—Cd1	99.4 (3)	C21—C16—C15	119.8 (4)
C22—O4—Cd1^i^	167.6 (3)	C16—C17—C18	120.6 (5)
C22—O4—Cd1	86.3 (3)	C16—C17—H17	119.7
Cd1^i^—O4—Cd1	106.03 (13)	C18—C17—H17	119.7
N1—C1—C2	123.0 (6)	C17—C18—C19	117.9 (5)
N1—C1—H1	118.5	C17—C18—H18	121.1
C2—C1—H1	118.5	C19—C18—H18	121.1
C3—C2—C1	120.2 (6)	C20—C19—C18	121.9 (5)
C3—C2—H2	119.9	C20—C19—Br1	120.1 (4)
C1—C2—H2	119.9	C18—C19—Br1	118.0 (4)
C2—C3—C4	116.3 (6)	C19—C20—C21	119.4 (5)
C2—C3—C6	123.1 (7)	C19—C20—H20	120.3
C4—C3—C6	120.5 (7)	C21—C20—H20	120.3
C3—C4—C5	120.9 (6)	C20—C21—C16	119.9 (5)
C3—C4—H4	119.6	C20—C21—H21	120.1
C5—C4—H4	119.6	C16—C21—H21	120.1
N1—C5—C4	122.1 (6)	O3—C22—O4	121.8 (5)
N1—C5—H5	119.0	O3—C22—C23	118.3 (4)
C4—C5—H5	119.0	O4—C22—C23	119.9 (4)
C7—C6—C3	117.9 (8)	C24—C23—C28	120.2 (5)
C7—C6—C7^i^	52.5 (12)	C24—C23—C22	120.5 (5)
C3—C6—C7^i^	114.6 (8)	C28—C23—C22	119.3 (4)
C7—C6—H6A	107.8	C23—C24—C25	121.0 (5)
C3—C6—H6A	107.8	C23—C24—H24	119.5
C7^i^—C6—H6A	58.9	C25—C24—H24	119.5
C7—C6—H6B	107.8	C26—C25—C24	117.6 (5)
C3—C6—H6B	107.8	C26—C25—H25	121.2
C7^i^—C6—H6B	137.6	C24—C25—H25	121.2
H6A—C6—H6B	107.2	C25—C26—C27	123.5 (5)
C6—C7—C6^i^	117.0 (9)	C25—C26—Br2	119.4 (4)
C6—C7—H7A	108.1	C27—C26—Br2	117.2 (4)
C6^i^—C7—H7A	108.0	C26—C27—C28	118.1 (5)
C6—C7—H7B	108.0	C26—C27—H27	121.0
C6^i^—C7—H7B	108.0	C28—C27—H27	121.0
H7A—C7—H7B	107.3	C23—C28—C27	119.6 (5)
N2—C8—C9	123.4 (6)	C23—C28—H28	120.2
N2—C8—H8	118.3	C27—C28—H28	120.2
C9—C8—H8	118.3		
			
N2—Cd1—N1—C1	−11.9 (10)	C4—C3—C6—C7	−119.0 (12)
O1—Cd1—N1—C1	113.0 (5)	C2—C3—C6—C7^i^	116.5 (12)
O3—Cd1—N1—C1	−160.7 (5)	C4—C3—C6—C7^i^	−60.0 (12)
O4^i^—Cd1—N1—C1	−34.6 (5)	C3—C6—C7—C6^i^	65.9 (15)
O2—Cd1—N1—C1	58.3 (5)	C7^i^—C6—C7—C6^i^	−34.9 (13)
O4—Cd1—N1—C1	−108.4 (5)	C12—N2—C8—C9	1.7 (9)
C15—Cd1—N1—C1	85.6 (5)	Cd1—N2—C8—C9	−175.4 (5)
N2—Cd1—N1—C5	165.2 (7)	N2—C8—C9—C10	2.3 (11)
O1—Cd1—N1—C5	−69.9 (5)	C8—C9—C10—C11	−6.3 (11)
O3—Cd1—N1—C5	16.4 (5)	C8—C9—C10—C13	174.0 (6)
O4^i^—Cd1—N1—C5	142.5 (5)	C9—C10—C11—C12	6.4 (11)
O2—Cd1—N1—C5	−124.6 (5)	C13—C10—C11—C12	−173.9 (6)
O4—Cd1—N1—C5	68.7 (5)	C8—N2—C12—C11	−1.7 (9)
C15—Cd1—N1—C5	−97.3 (5)	Cd1—N2—C12—C11	175.4 (5)
N1—Cd1—N2—C8	11.7 (10)	C10—C11—C12—N2	−2.5 (11)
O1—Cd1—N2—C8	−113.8 (5)	C11—C10—C13—C14	115.9 (10)
O3—Cd1—N2—C8	160.4 (5)	C9—C10—C13—C14	−64.4 (12)
O4^i^—Cd1—N2—C8	34.3 (5)	C11—C10—C13—C14^i^	54.7 (10)
O2—Cd1—N2—C8	−59.3 (5)	C9—C10—C13—C14^i^	−125.6 (9)
O4—Cd1—N2—C8	108.1 (5)	C10—C13—C14—C13^i^	−63.9 (13)
C15—Cd1—N2—C8	−86.9 (5)	C14^i^—C13—C14—C13^i^	32.8 (11)
N1—Cd1—N2—C12	−165.3 (7)	Cd1—O1—C15—O2	6.5 (6)
O1—Cd1—N2—C12	69.2 (4)	Cd1—O1—C15—C16	−173.8 (4)
O3—Cd1—N2—C12	−16.6 (5)	Cd1—O2—C15—O1	−6.1 (5)
O4^i^—Cd1—N2—C12	−142.7 (5)	Cd1—O2—C15—C16	174.2 (4)
O2—Cd1—N2—C12	123.7 (4)	N1—Cd1—C15—O1	91.8 (3)
O4—Cd1—N2—C12	−68.9 (4)	N2—Cd1—C15—O1	−99.7 (3)
C15—Cd1—N2—C12	96.1 (5)	O3—Cd1—C15—O1	−5.5 (4)
N1—Cd1—O1—C15	−91.5 (3)	O4^i^—Cd1—C15—O1	178.1 (3)
N2—Cd1—O1—C15	79.1 (3)	O2—Cd1—C15—O1	174.0 (5)
O3—Cd1—O1—C15	174.9 (3)	O4—Cd1—C15—O1	−23.0 (7)
O4^i^—Cd1—O1—C15	−3.1 (5)	N1—Cd1—C15—O2	−82.3 (3)
O2—Cd1—O1—C15	−3.4 (3)	N2—Cd1—C15—O2	86.3 (3)
O4—Cd1—O1—C15	171.0 (3)	O1—Cd1—C15—O2	−174.0 (5)
N1—Cd1—O2—C15	99.9 (3)	O3—Cd1—C15—O2	−179.5 (3)
N2—Cd1—O2—C15	−91.0 (3)	O4^i^—Cd1—C15—O2	4.0 (4)
O1—Cd1—O2—C15	3.3 (3)	O4—Cd1—C15—O2	162.9 (4)
O3—Cd1—O2—C15	0.7 (4)	O1—C15—C16—C17	179.8 (5)
O4^i^—Cd1—O2—C15	−176.5 (3)	O2—C15—C16—C17	−0.5 (8)
O4—Cd1—O2—C15	−160.5 (5)	O1—C15—C16—C21	−0.1 (7)
N1—Cd1—O3—C22	86.1 (4)	O2—C15—C16—C21	179.6 (5)
N2—Cd1—O3—C22	−88.0 (4)	C21—C16—C17—C18	−1.5 (9)
O1—Cd1—O3—C22	−177.5 (4)	C15—C16—C17—C18	178.5 (5)
O4^i^—Cd1—O3—C22	1.2 (4)	C16—C17—C18—C19	2.0 (9)
O2—Cd1—O3—C22	−175.3 (3)	C17—C18—C19—C20	−1.7 (9)
O4—Cd1—O3—C22	−0.8 (3)	C17—C18—C19—Br1	179.3 (5)
C15—Cd1—O3—C22	−175.0 (3)	C18—C19—C20—C21	0.9 (9)
N1—Cd1—O4—C22	−94.4 (3)	Br1—C19—C20—C21	179.9 (4)
N2—Cd1—O4—C22	97.1 (3)	C19—C20—C21—C16	−0.4 (8)
O1—Cd1—O4—C22	5.7 (4)	C17—C16—C21—C20	0.7 (8)
O3—Cd1—O4—C22	0.8 (3)	C15—C16—C21—C20	−179.4 (5)
O4^i^—Cd1—O4—C22	−177.6 (3)	Cd1—O3—C22—O4	1.5 (6)
O2—Cd1—O4—C22	165.7 (5)	Cd1—O3—C22—C23	−179.1 (4)
C15—Cd1—O4—C22	21.3 (7)	Cd1^i^—O4—C22—O3	179.1 (14)
N1—Cd1—O4—Cd1^i^	85.51 (17)	Cd1—O4—C22—O3	−1.3 (5)
N2—Cd1—O4—Cd1^i^	−83.04 (17)	Cd1^i^—O4—C22—C23	0(2)
O1—Cd1—O4—Cd1^i^	−174.44 (17)	Cd1—O4—C22—C23	179.3 (4)
O3—Cd1—O4—Cd1^i^	−179.3 (2)	O3—C22—C23—C24	177.4 (5)
O4^i^—Cd1—O4—Cd1^i^	2.3 (2)	O4—C22—C23—C24	−3.1 (8)
O2—Cd1—O4—Cd1^i^	−14.4 (7)	O3—C22—C23—C28	−2.7 (7)
C15—Cd1—O4—Cd1^i^	−158.8 (5)	O4—C22—C23—C28	176.8 (5)
C5—N1—C1—C2	−3.2 (11)	C28—C23—C24—C25	−1.4 (8)
Cd1—N1—C1—C2	174.0 (6)	C22—C23—C24—C25	178.5 (5)
N1—C1—C2—C3	−0.5 (13)	C23—C24—C25—C26	−0.6 (8)
C1—C2—C3—C4	2.8 (13)	C24—C25—C26—C27	2.3 (9)
C1—C2—C3—C6	−173.9 (7)	C24—C25—C26—Br2	−177.1 (4)
C2—C3—C4—C5	−1.5 (13)	C25—C26—C27—C28	−1.8 (9)
C6—C3—C4—C5	175.2 (7)	Br2—C26—C27—C28	177.6 (4)
C1—N1—C5—C4	4.4 (10)	C24—C23—C28—C27	1.9 (8)
Cd1—N1—C5—C4	−172.8 (6)	C22—C23—C28—C27	−178.0 (5)
C3—C4—C5—N1	−2.1 (13)	C26—C27—C28—C23	−0.4 (8)
C2—C3—C6—C7	57.5 (14)		

Symmetry codes: (i) *y*, *x*, −*z*+1.
